# The Effect of the Doping Amount on Electroelastic Coupled-Wave Scattering and Dynamic Stress Concentration around Defects in BNT Doped FN Materials

**DOI:** 10.3390/ma15165781

**Published:** 2022-08-21

**Authors:** Jiawei Fan, Chuanping Zhou, Junqi Bao, Huawei Ji, Yongping Gong, Weihua Zhou, Jiang Lin

**Affiliations:** 1 School of Mechanical Engineering, Hangzhou Dianzi University, Hangzhou 310018, China; 2 School of Mechatronics Engineering, University of Electronic Science and Technology of China, Chengdu 611731, China; 3 Hangzhou Changchuan Technology Co., Ltd., Hangzhou 310018, China; 4 College of Electrical Engineering, Zhejiang University, Hangzhou 310027, China

**Keywords:** ferrum niobium ion, electroelastic wave, triangular defect, dynamic stress concentration factor, FN doping fraction

## Abstract

Sodium bismuth titanate (Bi_0.5_Na_0.5_TiO_3_, BNT) has attracted much attention because of its excellent dielectric, piezoelectric and electromechanical properties. The microstructure of sodium bismuth titanate-doped ferrum niobium material (Bi_0.5_Na_0.5_TiO_3_ doped (Fe_0.5_Nb_0.5_)^4+^, BNT-*x*FN) shows a triangle as its typical defect shape. Since piezoelectric devices usually operate under dynamic loads, they fail easily owing to dynamic stress concentration or dynamic fracture. Elastic waves can simulate many types of dynamic loads, and the dynamic stress concentration caused by an anti-plane shear wave is the basis for the calculation of the stress field strength factor of type Ⅲ-dynamic fractures. In this study, the electroelastic coupled-wave diffraction and dynamic stress concentration of BNT-*x*FN materials with triangular defects under the incidence of anti-plane shear waves were studied. Maxwell equations are decoupled by auxiliary functions, and the analytical solutions of the elastic wave field and electric field are obtained. Based on the conformal mapping method, the triangle defect was mapped to the unit circle defect, and the dynamic stress concentration coefficient around the triangle defect was obtained by calculating the undetermined mode coefficients in the expression through boundary conditions. The numerical calculation shows that the size of the triangular hole, the frequency of the applied mechanical load, the incidence angle of mechanical load and the amount of FN doping have a great influence on the stress concentration of BNT-*x*FN materials.

## 1. Introduction

Piezoelectric materials exhibit a mechanical response to electrical inputs, or an electrical response to mechanical inputs, which makes them strong candidates for applications as sensors, ultrasonic transducers and piezoelectric actuators, etc. [1]. Sodium bismuth titanate (Bi_0.5_Na_0.5_TiO_3_) is a kind of important perovskite type lead-free ferroelectric and piezoelectric material with excellent dielectric, piezoelectric and electromechanical properties. Zhang et al. discovered the extremely large electrostrain-induced properties for the first time in the study of the system (BNT-BT-KNN), with the maximum unipolar strain as high as 0.45%. BNT matrix materials are considered to be promising to replace the lead-based materials in commercial piezoelectric actuators. However, due to the temperature stability, the driving electric field and fatigue resistance characteristics will limit their application, so the normal ferroelectrics are doped FN in order to improve the material structure and related electrical properties. In the existing studies, the doping of FN has made a significant disruption of the long-range ferroelectric order of BNT, resulting in its high unipolar strain and piezoelectric constant, which plays a significant role in regulating the electrical properties of ceramics and is suitable for making large-displacement lead-free piezoelectric brakes, etc. [2].

However, defects usually occur in mechanical parts and engineering structures during fabrication and service process, such as dislocations, cracks, holes, and inclusions, which may seriously affect the service life and the performance of those piezoelectric devices and structures. Especially under various dynamic loading and severe conditions (high speed, high temperature and vibration, etc.) at the discontinuous interface or apertures and openings, the dynamic stress concentration may increase markedly, which results in structural strength reduction, structural fatigue or fracture [3,4,5,6]. Therefore, it is of practical significance to analyze the dynamic stress of the problem of cracked holes under the coupling of a force field and electric field.

From the available literature, a great number of numerical, experimental and theoretical studies on the piezomagnetic/piezoelectric materials with cracked holes have been carried out to improve the strength and stability of piezomagnetic materials and structures [7,8,9,10,11]. Jiao et al. [7] studied the diffraction problems of elastic waves in a piezoelectric half-space and piezoelectric half-space sandwiched in gradient blocks by using the transfer matrix method. Liang et al. [8] studied the magneto-elastic coupling effect of infinite soft ferromagnetic materials with cracks. The magneto-elastic coupling interface of soft magnetic materials with cracks under uniform magnetic induction was studied [9]. For cracks in functional composites with piezomagnetic, piezoelectric and magnetoelectric coupling effects, Wang and Mai [10] provided a theoretical method to calculate the strength factor, magnetic induction and electric displacement near the crack tip. Cao et al. [11] studied the propagation characteristics of Lamb waves in functionally graded piezoelectric/piezomagnetic composites with continuous changes along the thickness direction. The dispersion equations under different boundary conditions are given. The influence of parameter variation on a dispersion curve and cutoff frequency in an electromagnetic field is discussed in detail. Wang et al. [12] studied the anti-plane problem of isotropic piezoelectric elastic solids with equilateral triangular holes with smooth vertices by constructing a new conformal mapping. Singh et al. [13] studied the propagation characteristics of Shear Horizontal Waves (SH waves) in two semi-infinite voltage magnetic materials and obtained different forms of the explicit nonuniformity of the dispersion relation. Rogowski [14] considered the problems of two asymmetric edge cracks originating from electro-magneto-elastic loads of elliptical holes in coupled media. Wang and Gao [15] used Stroh’s formula to study the anti-plane problem of middle-edge triangular holes in transversely isotropic piezoelectric materials.

Wang and Gao [16] studied the mode III fracture problem of circular hole edge crack in an infinite piezoelectric body based on the complex variable method, and proposed a complex potential, field strength factor and energy release rate expression of the mode III fracture problem of circular hole edge crack in an infinite piezoelectric body based on the compound variable method. Tian et al. [17] analyzed the propagation of SH waves in a layered structure composed of functionally gradient piezoelectric layers and piezomagnetic half-space. The relationship between the gradient coefficient and the thickness of the medium layer in SH wave propagation is explained. Pang et al. [18] studied the propagation and position of positive and oblique waves in piezoelectric/piezomagnetic layered periodic structures based on the transfer matrix method and the stiffness matrix method. The corresponding dispersion curve, localization factor and response spectrum characteristics are obtained by calculation. Fang et al. [19] studied the surface/interface stress of dynamic stress around spherical non-uniformity under asymmetric dynamic loading. Guo et al. [20,21] obtained the exact solution for the anti-plane problem of two edge asymmetric cracks in an elliptical hole of piezoelectric material under the assumption of electrical impermeability and permeability. Gao and Noda [22] studied the anti-plane problem of infinite piezoelectric materials with arbitrary holes by using Faber series and the complex method. Due to the particularity of the actual working environment of materials and the severity of the defect shapes, the above research either lacks the simulation of different working environments or the analysis of specific material defect shapes. Therefore, in this paper, we choose to use incident waves in different directions and on triangular defects to analyze the mechanical properties of materials.

In this paper, the electroelastic coupling wave is used to simulate the vibration of the BNT-*x*FN material in different working environments, and the typical triangular defect structure observed under the microscope is used to characterize the defect shape. Meanwhile, the specific mechanical properties are characterized by the dynamic stress concentration factor (DSCF). [23] Firstly, the Maxwell equation and dynamic equation are used to establish the overall model, and then the elastic wave field and potential are described by the wave function expansion method. Among them, the specific coefficients of the diffraction field expansion of BNT-*x*FN can be determined by combining the boundary conditions. After substituting the specific coefficients, the analytical expression of DSCF can be obtained. The effects of the incident wave number, the doping amount of FN and the geometric parameters on DSCF around the aperture are analyzed and discussed.

## 2. Dynamics Equation and Maxwell Equation in BNT-xFN Materials and the Decoupling

**Figure 1:**
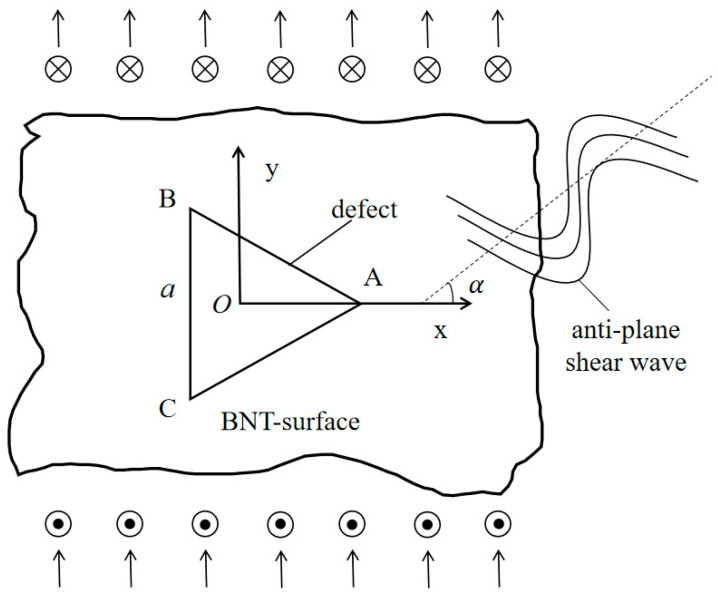
A triangular hole in BNT materials and the wave simulation.

A triangular hole embedded in the infinite BNT-*x*FN material is shown in Figure 1. The BNT-*x*FN material is set to be uniform and homogeneous. The anti-plane shear wave propagates in the direction of degrees to the x-axis in the infinite BNT-*x*FN material. In this case, the anti-plane dynamics equation and simplified Maxwell equation in BNT-*x*FN materials in a polar coordinate system are [23].

(1) $$\begin{array}{l} \frac{1}{r}\frac{\partial \tau_{\theta z}}{\partial \theta }+\frac{\partial \tau_{rz}}{\partial r}+\frac{\tau_{rz}}{r}=\rho \frac{\partial^{2}w}{\partial t^{2}}\\ \frac{\partial (rD_{r})}{\partial r}+\frac{\partial (D_{\theta })}{\partial \theta }=0 \end{array}$$

where $\tau_{\theta z},\tau_{\mathrm{rz}}$ are the shear stress components, ρ is the density, w is the displacement in the z-direction and $D_{r},\;D_{\theta }$ are the electric displacement intensities.

The constitutive relation of BNT-*x*FN materials with electric electromechanical coupling can be obtained:

(2) $$\begin{array}{l} \tau_{rz}=c_{44}\frac{\partial w}{\partial r}+e_{15}\frac{\partial \phi }{\partial r}\\ \tau_{\theta z}=c_{44}\frac{1}{r}\frac{\partial w}{\partial \theta }+e_{15}\frac{1}{r}\frac{\partial \phi }{\partial \theta }\\ D_{r}=e_{15}\frac{\partial w}{\partial r}-\varepsilon_{11}\frac{\partial \phi }{\partial r}\\ D_{\theta }=e_{15}\frac{1}{r}\frac{\partial w}{\partial \theta }-\varepsilon_{11}\frac{1}{r}\frac{\partial \phi }{\partial \theta } \end{array}$$

where $c_{44}$ is the elastic stiffness constant of BNT-*x*FN materials, $e_{15}$ is the piezoelectric stress constant, $\varepsilon_{11}$ is the dielectric constant and φ is the potential in materials.

By substituting Equation (2) into Equation (1), the following expressions are given:

(3) $$\begin{array}{l} c_{44}(\frac{1}{r^{2}}\frac{\partial^{2}w}{\partial \theta^{2}}+\frac{\partial^{2}w}{\partial r^{2}}+\frac{1}{r}\frac{\partial w}{\partial r})+e_{15}(\frac{1}{r^{2}}\frac{\partial^{2}\varphi }{\partial \theta^{2}}+\frac{\partial^{2}\varphi }{\partial r^{2}}+\frac{1}{r}\frac{\partial \varphi }{\partial r})=\rho \frac{\partial^{2}w}{\partial t^{2}}\\ e_{15}(r\frac{\partial^{2}w}{\partial r^{2}}+\frac{1}{r}\frac{\partial^{2}w}{\partial \theta^{2}}+\frac{\partial w}{\partial r})-\varepsilon_{11}(r\frac{\partial^{2}\varphi }{\partial r^{2}}+\frac{1}{r}\frac{\partial^{2}\varphi }{\partial \theta^{2}}+\frac{\partial \varphi }{\partial r})=0 \end{array}$$

The governing equations of displacement and electric potential need to be decoupled and the decoupling function is introduced [24]:

(4) $$\gamma =\varphi -\frac{e_{15}}{\varepsilon_{11}}w$$

Using a Laplace operator to simplify the equation, the wave and Laplace equation are obtained:

(5) $$\begin{array}{l} \nabla^{2}w=\frac{1}{c_{s}{}^{2}}\frac{\partial^{2}w}{\partial t^{2}}\\ \begin{array}{cc} & \nabla^{2}\gamma =0 \end{array} \end{array}$$

where ${\;c}_{s}=\sqrt{\chi /\rho_{0}}$ is the propagation velocity of anti-plane shear waves.

## 3. Total Wave Field in BNT-*x*FN Materials

Consider an anti-plane shear wave propagating at an angle of degrees to the x-axis. In the polar coordinate system (*r*, *θ*), the incident waves can be expanded as

(6) $$\begin{array}{l} w^{i}=w_{0}e^{i\left[k(x\cos\alpha +y\sin\alpha )-\omega t\right]}\\ w^{i}=\frac{e_{15}}{\varepsilon_{11}}w_{0}e^{i\left[k(x\cos\alpha +y\sin\alpha )-\omega t\right]} \end{array}$$

According to Equation (5), the scattered field caused by the aperture in BNT-*x*FN materials can be given as

(7) $$\begin{array}{l} w^{s}=\sum_{n=-\infty }^{\infty }A_{n}H_{n}^{\left(1\right)}(kr)e^{in\theta }\\ \gamma =\sum_{n=0}^{\infty }B_{n}k^{-n}r^{-n}e^{in\theta }\\ \varphi^{s}=\frac{e_{15}}{\varepsilon_{11}}w^{s}+\gamma \end{array}$$

$A_{n}$ and $B_{n}$ are the undetermined coefficients used to describe the scattered elastic wave field and the scattered electric field, while $H_{n}^{\left(1\right)}\left(\cdot \right)$ is the 3rd-order Bessel function and $k=\omega /c_{s}$ is the incident wave number.

By superimposing the incident field, the scattered field and the reflected field, the electroelastic field in BNT-*x*FN materials can be expressed as

(8) $$\begin{array}{l} w^{t}=w^{i}+w^{s}\\ \varphi^{t}=\frac{e_{15}}{\varepsilon_{11}}(w^{i}+w^{s})+\gamma \end{array}$$

## 4. Boundary Conditions and Refracted Mode Coefficients

In order to solve the problem in Figure 1, the following two new variables are introduced:

(9) $$\begin{array}{l} \zeta =x+iy\\ \bar{\zeta }=x-iy \end{array}$$

**Figure 2:**
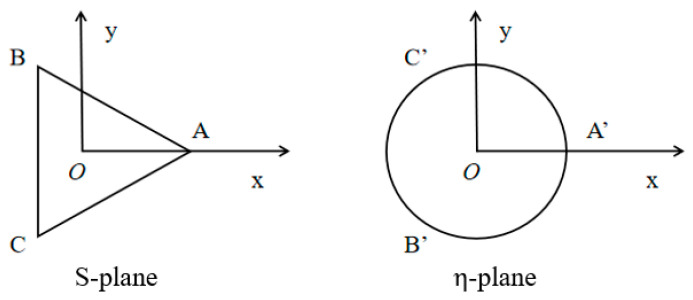
Conformal mapping.

The shape before and after the change is shown in Figure 2, the S-plane can be mapped to the  η−plane by using the conformal transformation [25]

(10) $$\zeta =\Omega (\eta )=R\left[\left(\frac{1}{\eta }\right)-\frac{1}{3}\cdot {\left(\frac{1}{\eta }\right)}^{-2}+\frac{1}{45}\cdot {\left(\frac{1}{\eta }\right)}^{-5}\right]$$

where *R* = 0.8381a.

Substituting the conformal transformation formula into the two-wave formula can be shown as

(11) $$\begin{array}{l} w^{\left(s\right)}=\sum_{n=-\infty }^{\infty }A_{n}{H_{n}}^{\left(1\right)}(k\left|\Omega (\eta )\right|){\left\{\frac{\Omega (\eta )}{\left|\Omega (\eta )\right|}\right\}}^{n}\\ \varphi^{\left(s\right)}=\frac{e_{15}}{\varepsilon_{11}}w^{\left(s\right)}+\sum_{n=0}^{\infty }B_{n}k^{-n}{\left(\bar{\Omega (\eta )}\right)}^{-n} \end{array}$$

(12) $$\begin{array}{l} w^{\left(i\right)}=w_{0}\sum_{n=-\infty }^{\infty }i^{n}J_{n}(k\left|\Omega (\eta )\right|){\left\{\frac{\Omega (\eta )}{\left|\Omega (\eta )\right|}\right\}}^{n}e^{-in\alpha }\\ \varphi^{\left(i\right)}=\frac{e_{15}}{\varepsilon_{11}}w^{\left(i\right)}=w_{0}\frac{e_{15}}{\varepsilon_{11}}\sum_{n=-\infty }^{\infty }i^{n}J_{n}(k\left|\Omega (\eta )\right|){\left\{\frac{\Omega (\eta )}{\left|\Omega (\eta )\right|}\right\}}^{n}e^{-in\alpha } \end{array}$$

The boundary conditions in plane can be written as

(13) $$\begin{array}{l} \tau_{\rho z}{|}_{\rho =a}=0\\ D_{\rho }{|}_{\rho =a}=D_{\rho }{}^{c}{|}_{\rho =a}=-\varepsilon_{11}\frac{\partial \varphi^{c}}{\partial \rho }{|}_{\rho =a}\\ \varphi^{\left(t\right)}{|}_{\rho =a}=\varphi^{c} \end{array}$$

By substituting the formula after conformal transformation into the boundary conditions, a determined infinite algebraic equation system can be formed:

(14) $$\sum_{n=-\infty }^{\infty }E_{n}X_{n}=E$$

Multiplying both ends of equation by $e^{-is\theta }$ and using the orthogonality of the function system obtains

(15) $$\sum_{n=-\infty }^{\infty }{Ε}_{\mathrm{ns}}X_{n}=E_{s}$$

According to Equation (15), we can derive the infinite system of linear equations for computing the mode coefficients.

## 5. Dynamic Stress Concentration Factor of BNK-xFN

According to the definition of DSCF, DSCF is the ratio of the hoop shear stress around the aperture to the maximum stress:

(16) $$DSCF=\left|\frac{\tau_{\theta z}}{\tau_{0}}\right|$$

where $\tau_{0}=w_{0}\chi k$ and

(17) $$\tau_{\theta z}=c_{44}\frac{1}{r}\frac{\partial w}{\partial \theta }+e_{15}\frac{1}{r}\frac{\partial \varphi }{\partial \theta }$$

According to the conformal transformation and the derivation rule, the derivation formula can be obtained as follows:

(18) $$\tau_{\theta z}=c_{44}i(\frac{\partial w}{\partial \zeta }e^{i\theta }-\frac{\partial w}{\partial \bar{\zeta }}e^{-i\theta })+e_{15}i(\frac{\partial \varphi }{\partial \zeta }e^{i\theta }-\frac{\partial \varphi }{\partial \bar{\zeta }}e^{-i\theta })$$

Using the derivation of the Bessel function, the final expression can be obtained:

(19) $$\frac{\partial w^{t}}{\partial \zeta }=\frac{k}{2}\sum_{n=-\infty }^{+\infty }\left[w_{0}i^{n}J_{n-1}(k\left|\Omega (\eta )\right|)e^{-in\alpha }+A_{n}H_{n-1}^{1}(k\left|\Omega (\eta )\right|)\right]{\left[\frac{\Omega (\eta )}{\left|\Omega (\eta )\right|}\right]}^{n-1}$$

(20) $$\frac{\partial w^{t}}{\partial \bar{\zeta }}=-\frac{k}{2}\sum_{n=-\infty }^{+\infty }\left[w_{0}i^{n}J_{n+1}(k\left|\Omega (\eta )\right|)e^{-in\alpha }+A_{n}H_{n+1}^{1}(k\left|\Omega (\eta )\right|)\right]{\left[\frac{\Omega (\eta )}{\left|\Omega (\eta )\right|}\right]}^{n+1}$$

(21) $$\frac{\partial \varphi^{t}}{\partial \zeta }=\frac{ke_{15}}{2\varepsilon_{11}}\sum_{n=-\infty }^{+\infty }\left[w_{0}i^{n}J_{n-1}(k\left|\Omega (\eta )\right|)e^{-in\alpha }+A_{n}H_{n-1}^{1}(k\left|\Omega (\eta )\right|)\right]{\left[\frac{\Omega (\eta )}{\left|\Omega (\eta )\right|}\right]}^{n-1}$$

(22) $$\frac{\partial \varphi^{t}}{\partial \bar{\zeta }}=-\frac{ke_{15}}{2\varepsilon_{11}}\sum_{n=-\infty }^{+\infty }\left[w_{0}i^{n}J_{n+1}(k\left|\Omega (\eta )\right|)e^{-in\alpha }+A_{n}H_{n+1}^{1}(k\left|\Omega (\eta )\right|)\right]{\left[\frac{\Omega (\eta )}{\left|\Omega (\eta )\right|}\right]}^{n+1}-\sum_{n=0}^{+\infty }nB_{n}k^{-n}{\bar{\Omega (\eta )}}^{-(n+1)}$$

Thus, the DSCF around the triangle aperture in the BNT-*x*FN material is expressed as

(23) $$\begin{array}{l} DSCF=\frac{k}{2}c_{44}i(1+\frac{e_{15}^{2}}{c_{44}\varepsilon_{11}})\sum_{n=-\infty }^{\infty }\{\frac{\eta }{a}[w_{0}i^{n}J_{n-1}(k\left|\Omega (\eta )\right|)e^{-in\alpha }+A_{n}H_{n-1}^{1}(k\left|\Omega (\eta )\right|)]{\left[\frac{\Omega (\eta )}{\left|\Omega (\eta )\right|}\right]}^{n-1}\\ +\frac{\bar{\eta }}{a}[w_{0}i^{n}J_{n+1}(k\left|\Omega (\eta )\right|)e^{-in\alpha }+A_{n}H_{n+1}^{1}(k\left|\Omega (\eta )\right|)]{\left[\frac{\Omega (\eta )}{\left|\Omega (\eta )\right|}\right]}^{n+1}\}+e_{15}i\sum_{n=0}^{+\infty }\frac{\bar{\eta }}{a}nB_{n}k^{-n}{\bar{\Omega (\eta )}}^{-(n+1)} \end{array}$$

## 6. Numerical Examples Simulation and Discussion

Using the above expression, we can accurately calculate the DSCF distribution around the triangular defect. Equation (23) is a continuous infinite series, but after the test calculation, the result meets the requirement of engineering accuracy after truncation at n greater than 10. For the BNT-*x*FN material mentioned above, when the FN doping amount *x* changes from 0 to 0.06, the corresponding piezoelectric constants change from 8.425$\;C\cdot m^{2}$ to 3.774$\;C\cdot m^{2}$, and the constants of other materials are relatively stable, as follows:${\;\varepsilon }_{11}=1.32\times \frac{10^{-8}\;C}{\mathrm{Vm}},c_{44}=15.3\times 10^{10}\;N\cdot m^{-2}$. In order to facilitate the analysis, the defect size and wave number were dimensionally processed. Then, the DSCF distribution at different incident angles was explored under the conditions of low frequency and high frequency, and the influence of different doping amounts on the mechanical properties of the materials was further studied. Firstly, the distribution of DSCF is discussed under different frequency conditions when the incident angle is 0.

**Figure 3:**
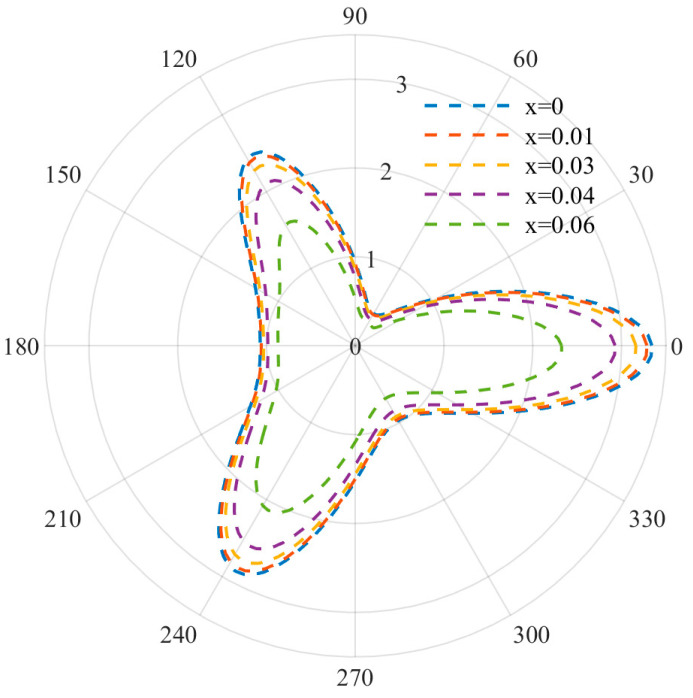
Distribution diagram of DSCD (*ka* = 0.8).

**Figure 4:**
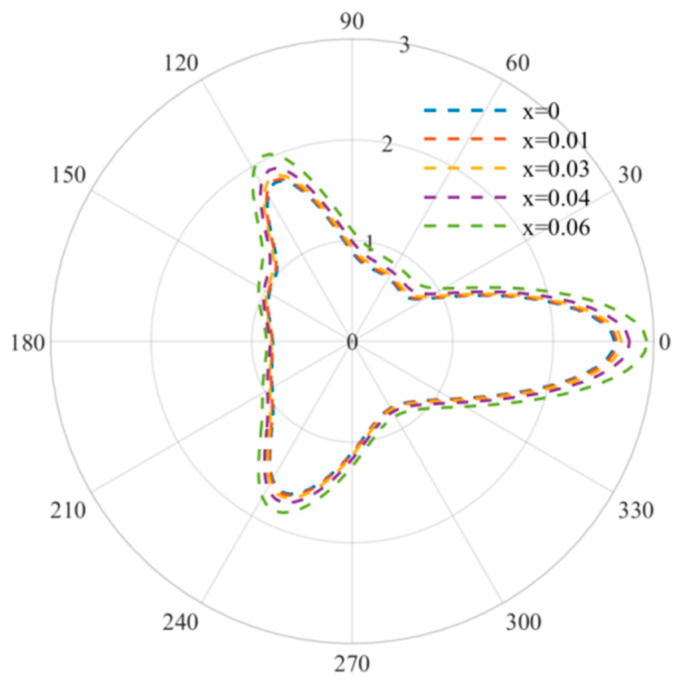
Distribution diagram of DSCD (*ka* = 1).

**Figure 5:**
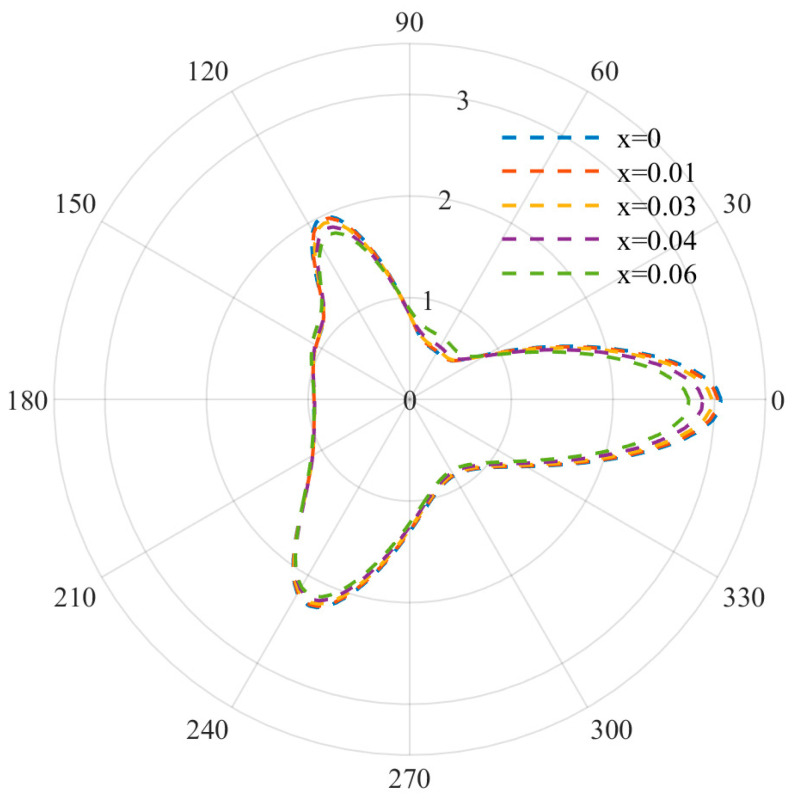
Distribution diagram of DSCD (*ka* = 1.2).

At relatively low frequencies, the DSCF distribution curves used to describe equilateral triangle defects are shown in Figure 3, Figure 4 and Figure 5. It can be observed that when the wave number is small, the distribution of DSCF has an obvious law: the stress distribution presents a triangular distribution, and the maximum value is obtained at ϑ=0,ϑ= π/3,ϑ=2π/3. Since the defect is triangular in shape, the stress concentration is easily achieved at the triangle, which is consistent with the engineering practice.

At this point, by observing the curve arrangement, it can be found that the different doping amount of FN has a great influence on the mechanical properties of the BNT material. When the frequency parameter *ka* = 0.8 and the FN doping amount is 0, the DSCF value at 0 is 1.3 times that of the FN doping amount 0.06. With an increase in the incident frequency, DSCF values with a different FN doping amount all began to decrease and were more evenly distributed. When *ka* = 1.2, the left DSCF decreased more gently. The above indicates that the mechanical properties of materials are easily affected with an increase in the doping amount at low frequency, and the doping amount should be designed reasonably.

**Figure 6:**
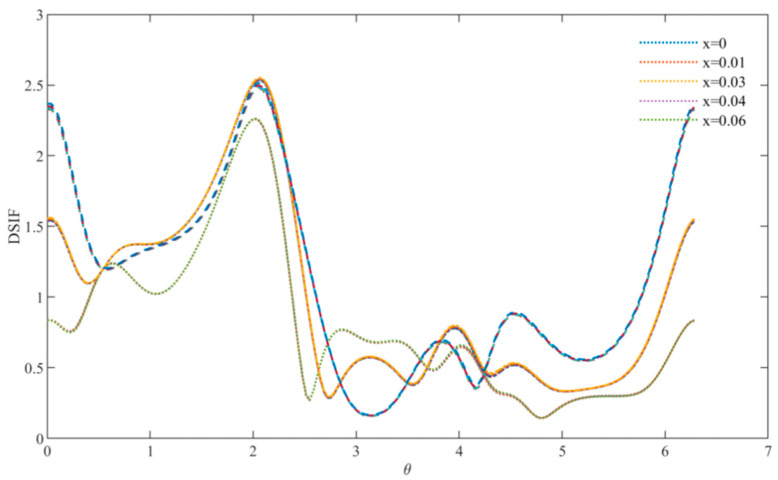
Comparison diagram of DSCF distribution at high frequency (*ka* = 3, 3.5, 4).

Figure 6 describes the DSCF distribution curve at regular triangle defects at relatively high frequencies, and the incident wave numbers are *k a* = 3, *ka* = 3.5 and *ka* = 4. At this point, the distribution of DSCF is no longer an obvious triangular distribution and reaches its maximum when ϑ  is about 2π/3, and the maximum value of DSCF decreases gradually with the increase in frequency. Different from the case of a relatively low frequency, the increase and decrease of DSCF are not obvious with the increase in the doping amount, which indicates that at a relatively high frequency, the FN doping amount has little influence on the mechanical properties of the overall material and can be added appropriately.

**Figure 7:**
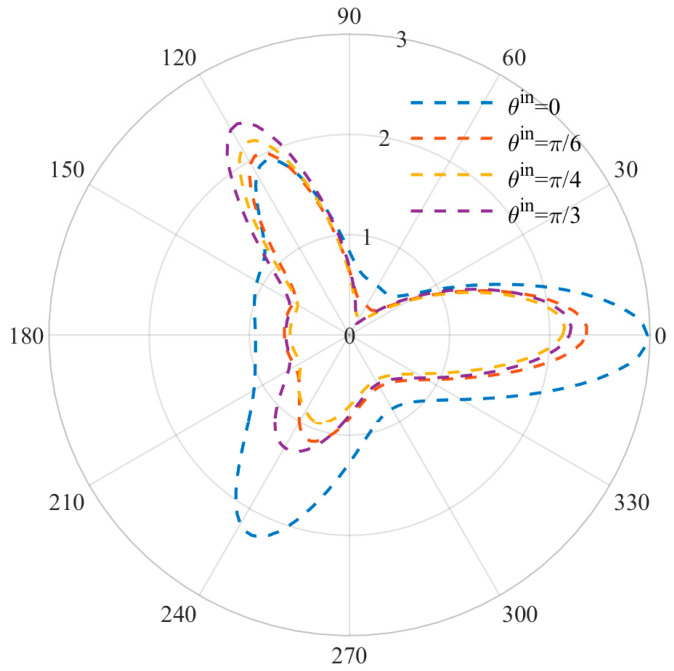
Distribution diagram of DSCF at different incident angles (*ka* = 1).

**Figure 8:**
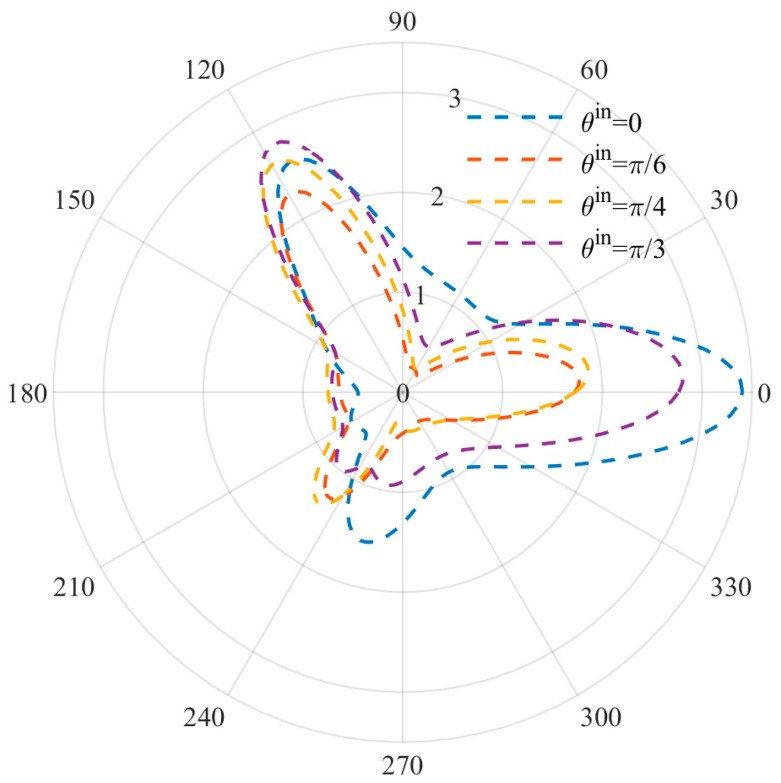
Distribution diagram of DSCF at different incident angles (*ka* = 2).

Figure 7 and Figure 8 show the DSCF distribution changes caused by different incident angles at low and high frequencies. The incidence angle of the incident wave is set as $\vartheta^{\mathrm{in}}=0,\;\pi /3,\pi /4,\pi /6\;$, and the wave number is set as *ka* = 1 and *ka* = 2. It can be observed that the positions of the three vertex angles where the extreme values were originally located at the incident angle $\vartheta^{\mathrm{in}}=0\;$ were all offset with the change of angle, but the offset was slight due to the stress concentration. At the same time, the DSCF values at $\vartheta^{\mathrm{in}}=0,\;2\pi /3$ reached the maximum when $\;\vartheta^{\mathrm{in}}=0$, while the DSCF values at ϑ=0  were small when $\vartheta^{\mathrm{in}}=0$, indicating that the incident angle has a certain influence on the DSCF values of the three vertices of the triangular defect, and the incident angle has a strengthening effect on the DSCF values of the corresponding vertex.

**Figure 9:**
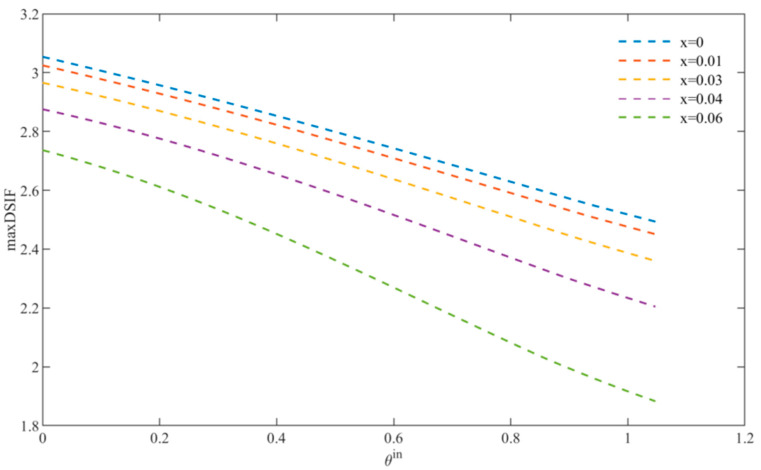
Variation of DSCF with incident angle.

In Figure 9, we can see that with the increasing incident angle, the maximum value of DSCF of BNT-*x*FN materials with different doping amounts gradually decreases, which means that the DSCF value can be greatly reduced by avoiding the incident wave directly shooting along the direction of triangular defects.

## 7. Conclusions

In this paper, we study the DSCF distribution and triangular defects in BNT-*x*FN materials under anti-plane shear waves. For numerical calculation, we use the wave function expansion method (WFE) and conformal mapping. Firstly, the triangular defect is mapped to the unit circle defect, and the displacement component and stress component are represented by the superposition of the wave function, where the wave function is the first and the third Bessel function. For the undetermined mode coefficients, the nonlinear equations are written by using the boundary conditions at the defects, and then the nonlinear equations are reduced to linear equations by orthogonalization. Then, the DSCF distribution of the BNT-*x*FN material with a triangular defect under a dynamic load simulation was studied from the following aspects: the size of the triangular defect hole, the distribution rule of the triangular defect, the frequency of the applied mechanical load and the amount of FN doping.

In order to obtain the general distribution law of DSCF under the working condition of the BNT-*x*FN material, the incident angle was fixed at 0 degrees, the relative incident frequency was from low frequency to high frequency and the FN doping amount was from 0 to 0.06 to simulate and solve the stress distribution of the BNT-*x*FN material. The distribution law was obtained in a triangular shape, that is, the DSCF value reaches the extremum at the three vertex angle positions. At the same time, the relative incident frequency and the FN doping amount were controlled to remain unchanged, and the influence of their increase and decrease on DSCF distribution was studied. When the incident wave number is fixed, the effects of the FN doping amount on the mechanical properties of BNT materials show different characteristics. When the number of incident waves is small, the increase or decrease of the FN doping amount has a significant effect on DSCF distribution, especially at an intermediate frequency, and an increase in the FN doping amount tends to lead to an increase in the DSCF value. With the increase of the wave number, the influence of the doping amount gradually disappears. Therefore, in different application environments, the doping amount proportion can be rationally selected according to this law.

When the doping amount is fixed, the relative incident wave number has little influence on DSCF at medium and low frequencies, but with an increase in the relative incident wave number, DSCF has a downward trend as a whole. Considering material optimization, the influence of the defect distribution law inside the BNT-*x*FN material on DSCF was transformed into a fixed defect distribution, and the incident angle was changed to simplify the problem. When the incident angle is at a certain angle, the triangular distribution law is weakened, and the DSCF value near the incident point increases, while the DSCF value far away from the incident point decreases. In addition, with an increase in the incident angle, the maximum DSCF value decreases under different doping amounts, which means that the overall distribution trend of triangular defects has a great influence on the overall mechanical properties of the material, and the DSCF value can be better reduced to avoid the triangular defects of more materials being shot into parallel by incident waves.

According to the above analysis, the BNT-*x*FN material can be better applied in different industrial environments, and with the increasing work requirements of the BNT-xFN material, this set of theories can provide a certain range of doping proportion selection for the optimal FN doping scheme.

## Institutional Review Board Statement

Not applicable.

## Data Availability

Not applicable.
